# P-2010. The Impact of Staff-Performed Reminder Call Timing on Appointment Attendance in an Infectious Diseases Clinic

**DOI:** 10.1093/ofid/ofaf695.2174

**Published:** 2026-01-11

**Authors:** Angelica M Chan, Mery Deeb, Hadeel Zainah

**Affiliations:** Brown University/Kent Hospital, Providence, RI; Brown University/Rhode Island Hospital, Providence, Rhode Island; Kent County-Memorial Hospital, Warwick, Rhode Island

## Abstract

**Background:**

Appointment attendance is an ongoing problem in many outpatient clinics. Despite adding staff-performed calls to automated calls in our Infectious Diseases (ID) clinic, we did not see benefit when staff-performed calls were done 1 day prior to appointments. We aimed to evaluate the effect of changing staff-performed calls to 2-3 days prior to the visit.

**Figure:**
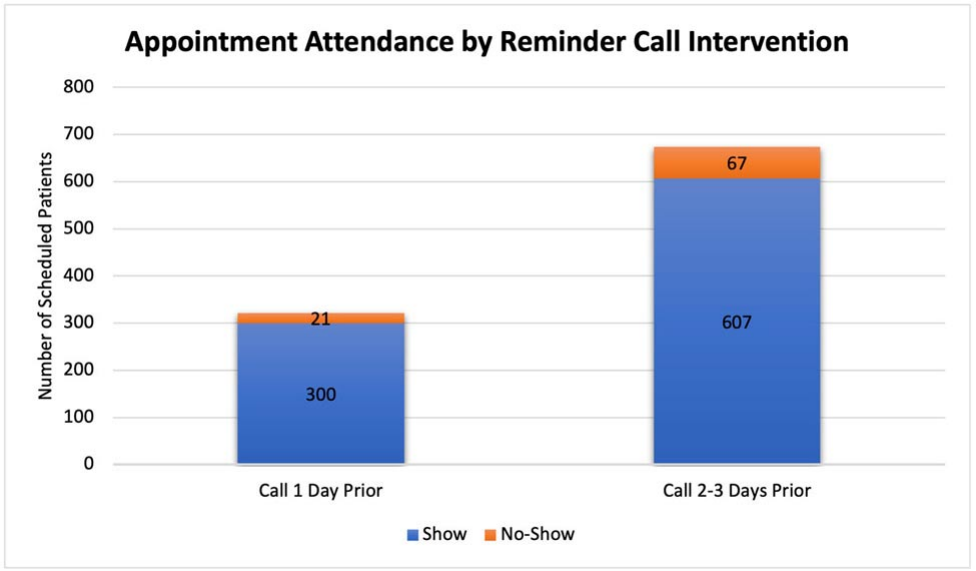

**Figure:**
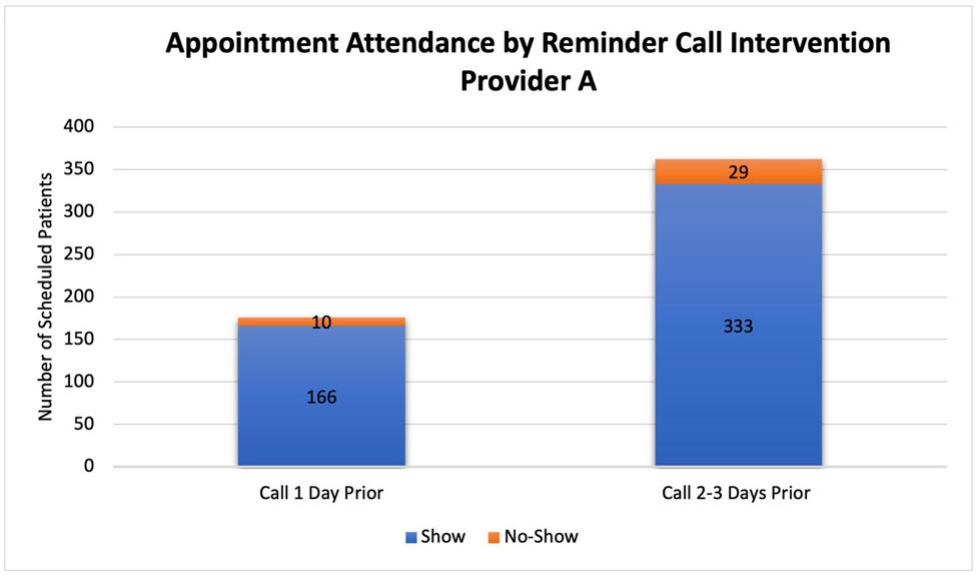

**Methods:**

This retrospective quality improvement project occurred in an ID clinic at a 359-bed Rhode Island community hospital. The clinic had 2 providers who see non-HIV patients. The staff performed reminder calls to patients who did not confirm appointments through the automated reminder calls. The timing of the reminder call was changed from 1 day to 2-3 days prior to appointment date to provide patients with more notice. The change started on May 26^th^, 2023. Data from a 6-month period before the change (November 1^st^, 2021-May 31^st^, 2022) were compared to data from a 10-month period after implementation (June 1^st^, 2023- March 31^st^, 2024).

**Figure:**
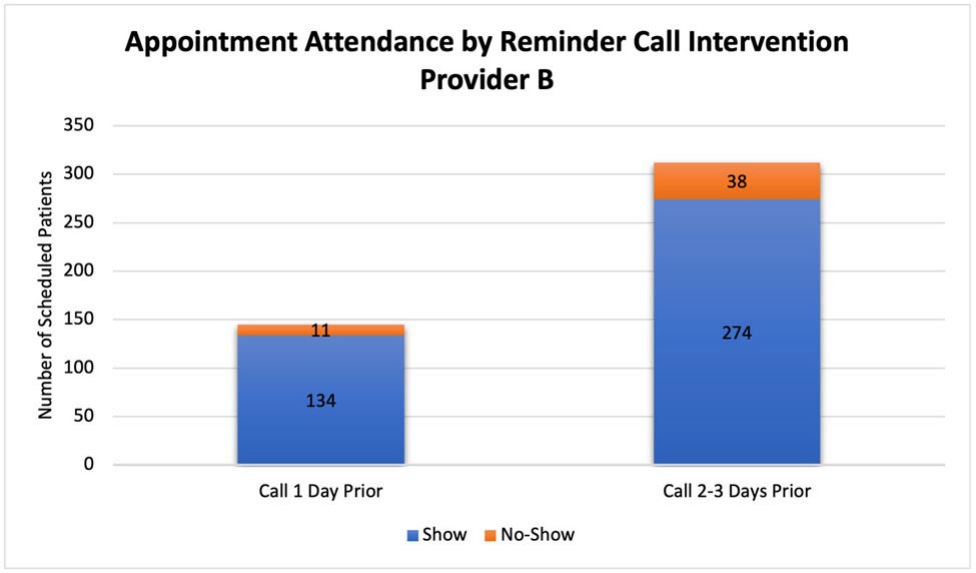

**Results:**

There were 321 patients scheduled when the reminder call was being done 1 day before appointment, 21 patients (6.54%) did not show up. There was a total of 607 patients after the change was implemented, of which 67 (11.03%) were no-shows. The no-show rate increased 4.49% after the call was performed 2-3 days earlier (P-value 0.094). The no-show rate for the first and second providers was 5.68% and 7.59% before the change and 8.7% and 13.87% after the change respectively. This indicated an increase of 3.02% (P-value 0.3791) and 6.28% (P-value 0.148) respectively. Insurance type and reason for the no-show were analyzed in the second period. Most of the no-shows had commercial insurance 79.1% (53 patients). Forty-eight no-shows had a known reason. The most common reason was the inability to deliver the reminder call to patients.

**Conclusion:**

Changing reminder calls from 1 day to 2-3 days prior to ID clinic appointment did not significantly impact no-show rates. No-show patients were most often insured by commercial plans. The most common reason for no-show was inability to deliver reminder calls. Further studies are needed to explore additional strategies to improve appointment adherence and related barriers.

**Disclosures:**

All Authors: No reported disclosures

